# Towards reduction of SAR in scaling up *in vivo* pulsed EPR imaging to larger objects

**DOI:** 10.1016/j.jmr.2018.12.011

**Published:** 2018-12-14

**Authors:** Randall Pursley, Ayano Enomoto, Haitao Wu, Jeffrey R. Brender, Thomas Pohida, Sankaran Subramanian, Murali C. Krishna, Nallathamby Devasahayam

**Affiliations:** a Signal Processing and Instrumentation Section, Computational Bioscience and Engineering Laboratory, Office of Intramural Research, National Institutes of Health, Bethesda, MD 20892, United States; b Radiation Biology Branch, Center for Cancer Research, National Cancer Institute, National Institutes of Health, Bethesda, MD 20892, United States; c Image Probe Development Center, National Heart, Lung, and Blood Institute, National Institutes of Health, Bethesda, MD 20892, United States; d Department of Biophysical Chemistry, Nagasaki International University, Japan; e Indian Institute of Technology, Madras, Chennai, India

**Keywords:** EPR, Stochastic excitation, Correlation, Bandpass sampling, Digital signal processing

## Abstract

An excessive RF power requirement is one of the main obstacles in the clinical translation of EPR imaging. The radio frequency (RF) pulses used in EPR imaging to excite electron spins must be very short to match their fast relaxation. With traditional pulse schemes and ninety degree flip angles, this can lead to either unsafe specific absorption rate (SAR) levels or unfeasibly long repetition times. In spectroscopy experiments, it has been shown that stochastic excitation and correlation detection can reduce the power while maintaining sensitivity but have yet to be applied to imaging experiments. Stochastic excitation is implemented using a pseudo-random phase modulation of the input stimulus. Using a crossed coil resonator assembly comprised of an outer saddle coil and an inner surface coil, it was possible to obtain a minimum isolation of ~50 dB across a 12 MHz bandwidth. An incident peak RF power of 5 mW was used to excite the system. The low background signal obtained from this resonator allowed us to generate images with 32 dB (>1000:1) signal-to-noise ratio (SNR) while exciting with a traditional pulse sequence in a phantom containing the solid paramagnetic probe NMP-TCNQ (N-methyl pyridinium tetracyanoquinodimethane). Using two different stochastic excitation schemes, we were able to achieve a greater than 4-fold increase in SNR at the same peak power and number of averages, compared to single pulse excitation. This procedure allowed imaging at significantly lower RF power levels than used in conventional EPR imaging system configurations. Similar techniques may enable clinical applications for EPR imaging by facilitating the use of larger RF coils while maintaining a safe SAR level.

## 1. Introduction

EPR imaging is receiving increased attention for *in vivo* applications in small animal molecular imaging research [1]. With several biologically compatible paramagnetic probes that can be administered *in vivo* at non-toxic doses [2], and implantable probes [3], EPR imaging provides information related to tissue redox and oxygenation status. EPR imaging in *in vivo* studies is typically conducted in the RF range of 300–1200 MHz. Nitroxyl probes are used to obtain tissue/organ redox status information by EPR imaging [4–6] while trityl radicals are used for oximetric imaging [2,7–11]. Non-invasive quantitative imaging of tumor pO_2_ is uniquely possible using EPR by determining the oxygen dependent line widths of paramagnetic probes such as Ox063. Such studies are being conducted in several murine models of human cancer in the field of drug discovery research [12].

EPR signals can be detected in the frequency domain using continuous-wave EPR (CW-EPR) or in the time-domain using pulsed Fourier transform EPR (FT-EPR) [4,10,13,14]. While CWEPR is useful in detecting signals from paramagnetic species with broad line widths [4–6,14], time-domain EPR is advantageous in the detection of narrow line species such as LiPc and trityls [2,7,10,15]. EPR imaging can be conducted in an analogous way as in MRI imaging with the use of field gradients. However, the gradients are managed differently. The phase memory times of the signals from ^1^H in MRI can be measured in hundreds of milliseconds, permitting the use of dynamic gradients for 2D frequency and phase encoding. In time domain-EPR such methods are found not practical because the phase memory times measured are in microseconds. Hence most EPR imaging is done using static field gradients [4,5,10,13]. The localization of the spins in EPR imaging is performed either by frequency-encoding followed by projection reconstruction, or phase-encoding followed by Fourier reconstruction [16,17]. The phase-encoding method was found to be advantageous, especially in time-domain EPR in terms of spatial and temporal resolutions, allowing the non-invasive study of tumor oxygenation dynamics [8,18]. Several EPR imaging studies have been conducted in tumor-bearing mice to better understand tumor physiology and it’s dynamics and its dependence on treatments such as radiation therapy, chemotherapy, or combination chemo-radiation therapy [11,19–23]. With the capabilities of EPR imaging in small animal research now well established in terms of data acquisition, image reconstruction, resonant structures, and spin probes, translating this modality to clinical applications is being investigated. While more readily accessible anatomical regions such as breast, prostate, and the oral cavity may be feasible with the present state of technology and available instrumentation, whole body applications may be limited by the restrictions on RF power exposure. The specific absorption rate associated with imaging in the 300 MHz range with the ~70 ns, 80 W pulses typically used in EPR imaging is around 0.7 W/kg. This SAR level is acceptable for local irradiation in humans but exceeds the limit for routine whole body imaging [24]. As 300 MHz frequency resonators capable of volumetric imaging become more common [9], there is renewed interest in low power pulse sequences that can meet FDA and International Electrotechnical Commission (IEC) guidelines with high duty cycles. To mitigate these restrictions, alternate strategies of EPR signal detection using lower RF power levels will be necessary [25,26].

**Fig. 1.**
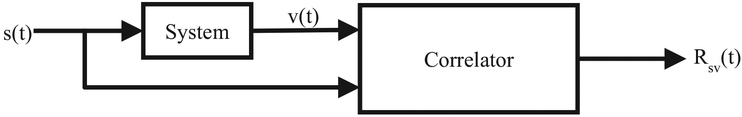
A pseudorandom noise excitation, s(t), stimulates the System and produces a response, v(t). The resulting correlation of these signals, R_sv_(t), is proportional to the impulse response of System.

**Fig. 2.**
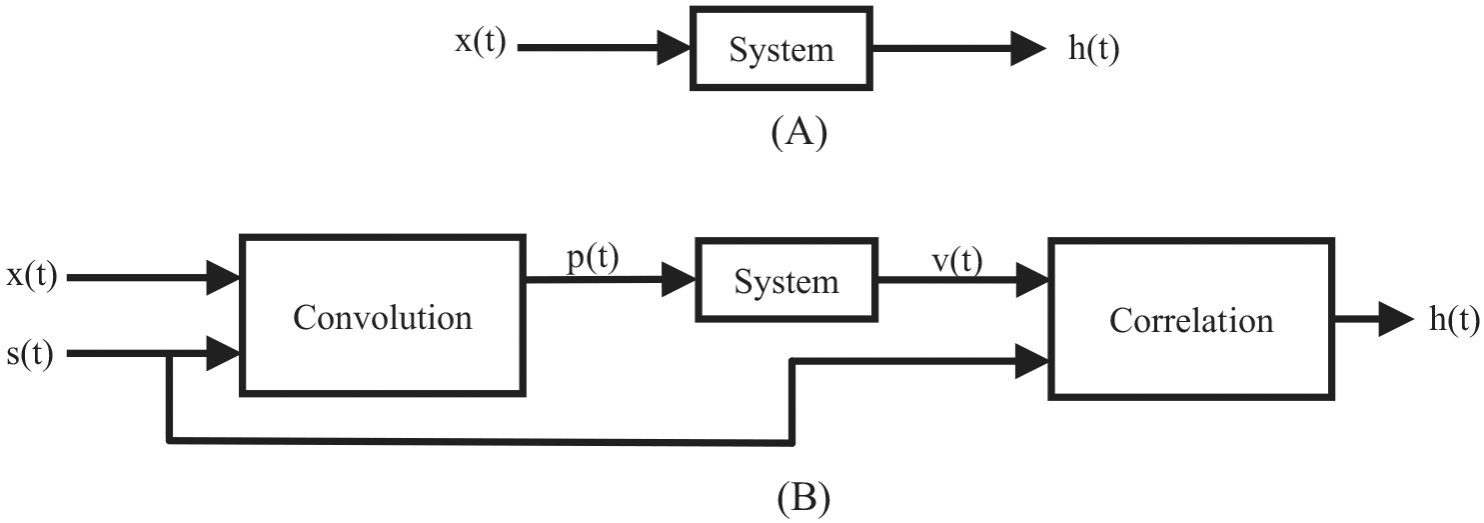
Comparison of a standard FT-EPR system to a system driven by a pulse train modulated with a pseudorandom sequence. (a) A RF pulse, x(t) is input into the system and the FID output, h(t), is observed. (b) A RF pulse, x(t) is convolved with a pseudorandom sequence, s(t), producing a sequence of modulated RF pulses, p(t). After correlating the output, v(t), with s(t), the FID output, h(t), is effectively observed.

Fig. 1 illustrates a generalized correlation spectroscopy system, where s(t) represents different input excitations, such as: pseudo-random noise or a stochastic sequence (i.e. a Hadamard or Frank sequence). Upon correlation of the system response, v(t), and the excitation, s(t), the response, R_sv_(t), is proportional to the impulse response of the System. The original stochastic excitation experiments used CW excitation to obtain the magnetic resonance signal. In the present study, the time-domain EPR method was utilized to avoid the limitations imposed by a CW excitation source. Fig. 2 illustrates the differences between a standard FT-EPR system and the current system.

In time-domain NMR spectroscopy, Ernst proposed the use of stochastic excitation by a binary Hadamard sequence-based pseudorandom phase modulation as a method to reduce peak RF power [27]. Under continuous stochastic excitation at low peak power, the spin system evolves in response to the phase modulation of the excitation. The correlation of the acquired response to this excitation and the originating stochastic phase modulation effectively yields the free induction decay (FID) that is typically generated by a single RF excitation pulse with higher peak power.

Because low power RF pulses are continually exciting the system, stochastic excitation has the additional advantage that the RF power is spread evenly through the duration of the experiment. In traditional time-domain EPR, RF pulses with high peak power excite the system at a much lower rate resulting in an uneven temporal distribution of RF power during an experiment. Pseudorandom excitation followed by correlation was implemented in EPR spectroscopy at 300 MHz to determine the SNR relative to conventional RF pulse methods using higher peak powers, and found to be comparable when using narrow line probes such as LiPc [28] following the early description at higher frequencies [29]. The use of Hadamard correlation spectroscopy in EPR at 300 MHz achieved an advantage of 13 dB in power reduction over the standard single RF pulse excitation modality.

Error correcting codes have been utilized in the communications industry for over 50 years. The original work in this field was focused on reducing bit errors during transmission of digital data in a noisy environment. Although the data acquired during EPR is analog in nature, the same techniques can be applied. The biphasic random excitation used in Hadamard correlation spectroscopy suffers from an imperfect impulse response cross-correlation with the response signal, as the circular autocorrelation function for lags other than zero is 1/N rather than zero. As a consequence, systematic noise is introduced into the signal in Hadamard biphasic sequences due to the finite sequence length [30].

Earlier reports implementing error correcting codes [31–33] by Blumich et al., addressed this problem with a stochastic polyphase Frank sequence with a circular autocorrelation function equal to the Kroenecker delta function [34] and demonstrated low power RF pulse excitation was possible at power levels six orders of magnitude lower than traditional single-pulse NMR modulated with comparable signal-to-noise. This prompted a study by Tseitlin et al. to implement a similar polyphase Frank sequence modulation scheme and detect EPR signals at a resonant frequency of 256 MHz. This work successfully detected resonance signals from the trityl spin probe despite the resonator recovery time being a significant fraction of the signal detection time. Signal detection was achieved with deuterated trityl radicals using a peak RF pulse power of 1.5 mW, which generated a flip angle of the magnetization vector of ~0.35°, which was cumulatively similar to that achieved with a single 90° pulse. The isolation of this resonant structure was sufficient to detect the long-lived induction signal from the trityl probe. These studies pointed to the feasibility of achieving signal detection with a sevenfold lower excitation than is typical [35,36].

Previous studies in stochastic excitation in EPR have focused on spectroscopy applications. Since imaging rather than spectroscopy has greater requirements in both power and signal, we compared the efficiency of both Hadamard and polyphasic stochastic excitation to the traditional single-pulse FT-EPR technique for imaging a solid NMP-TCNQ phantom at 300 MHz, using the same low power 5 mW excitation for each. At constant low peak power, the Hadamard and polyphasic sequences gave substantially higher SNR than traditional single pulse FT-EPR. The polyphasic sequence generated higher SNR then the Hadamard sequence. Results from this study demonstrate that low power EPR imaging is feasible and we are another step closer in translating EPR to clinical applications.

## 2. Materials and methods

### 2.1. Hardware description

**Fig. 3.**
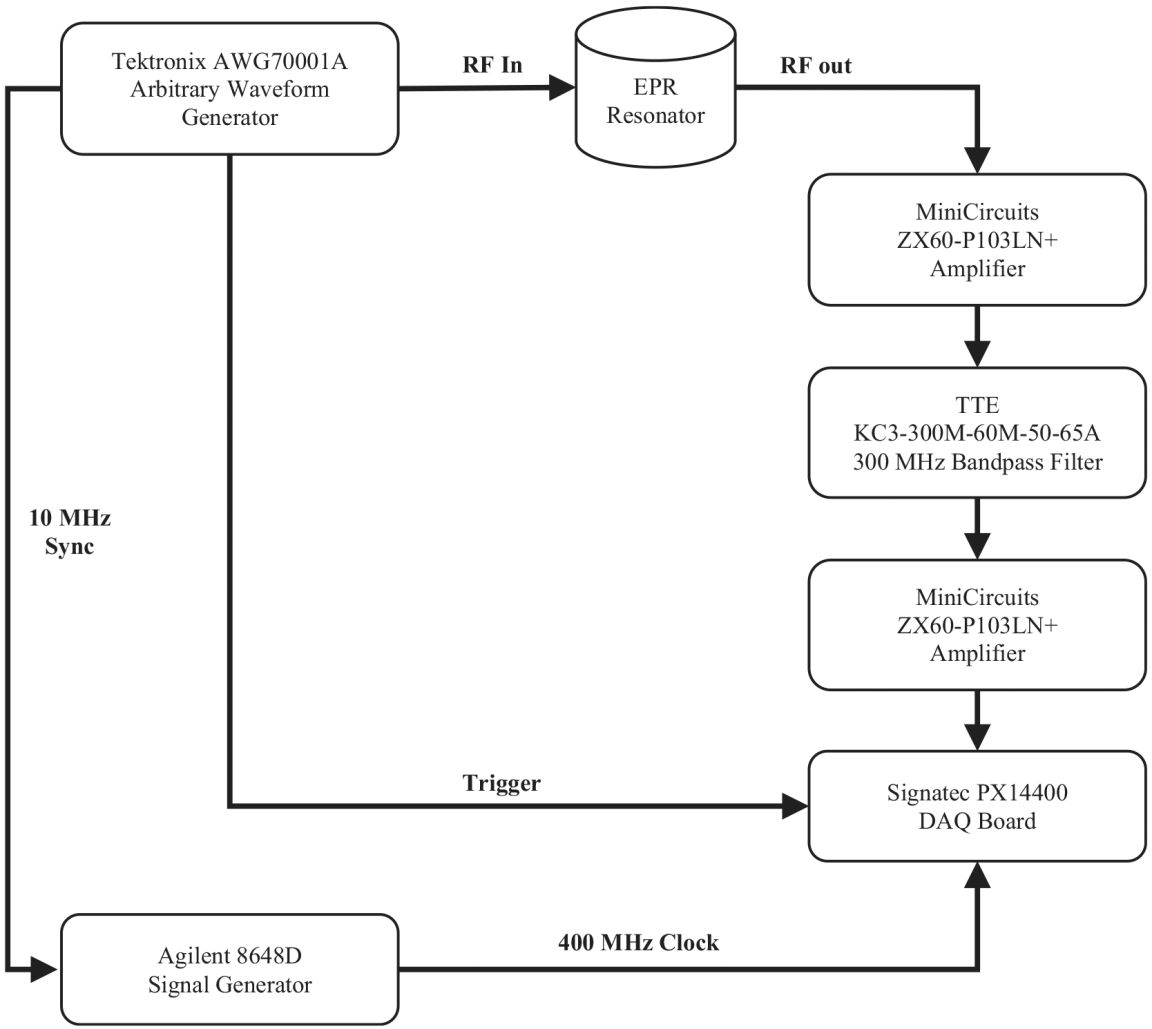
Block diagram of EPR system. In this experimental setup, a Tektronix AWG70001 arbitrary waveform generation is the RF excitation source. An Agilent 8648D is synchronized with the AWG to provide a synchronized clock to the Signatec PX14400 data acquisition board. The RF excitation is input into a custom in-house EPR cross-coil resonator. The RF response from the resonator is amplified and filtered before being acquired by the data acquisition board.

An existing 300 MHz time-domain EPR spectrometer was modified for these experiments. The hardware configuration for this system uses only commercial off-the-shelf equipment for signal generation and acquisition as shown in Fig. 3. The transmit section consists of the Tektronix AWG70001A arbitrary waveform generator (AWG) which can be configured to generate customized excitation signals and synchronized trigger signals. The length of the excitation waveforms was up to 184,320 points with 8-bit resolution. The output clock rate was 12 GS/s. A digital output was used to trigger an acquisition. A 10 MHz reference signal was used to synchronize the acquisition clock to the excitation clock to allow the implementation of bandpass sampling and accurate sampling relative to RF excitation.

**Fig. 4.**
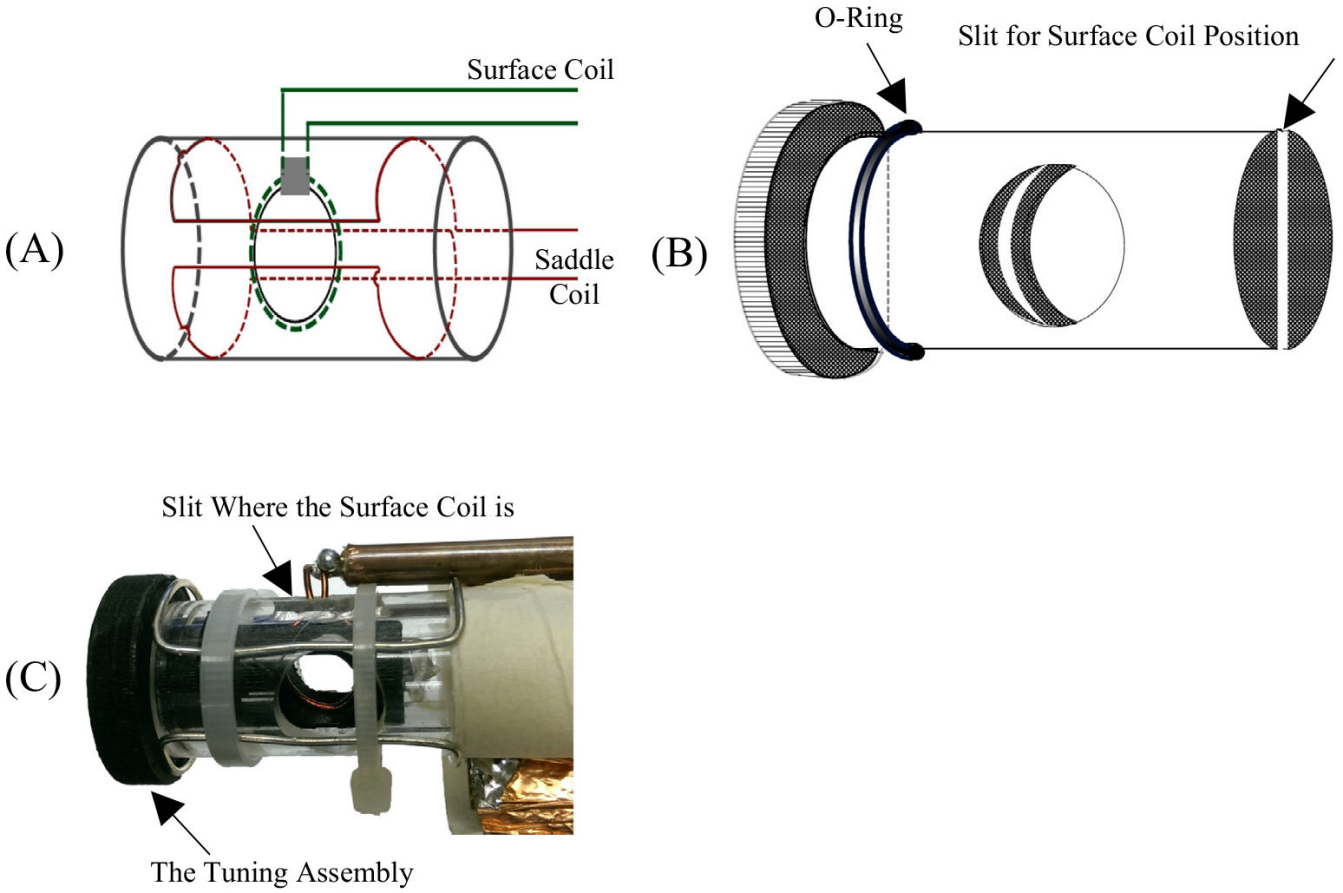
(A) Schematic of a crossed resonator with saddle transmit and an inner surface coil receive pair. (B) The tuning assembly. The mechanism is of a PVC insert fabricated with a slit in the middle. The diameter of the insert is the inner diameter of the Lucite tube. The slit width is equal to the diameter of the surface coil wire. In the assembly, an O-ring is positioned in such a way, once the tuning assembly is inserted, it will hold tightly. Since the surface coil is inside the slit, the position can be changed by tuning the assembly. (C) The actual resonator assembly.

**Fig. 5.**
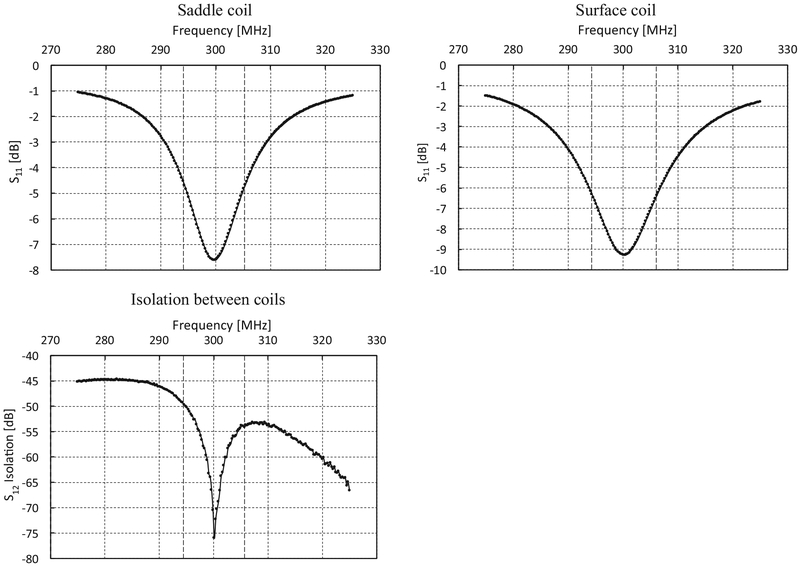
Frequency response of each coil and the measured isolation between the two coils with a center frequency of ~300 MHz and a 3 dB bandwidth of ~12 MHz for each coil. The isolation ranges from ~ 50 dB at the bandwidth boundaries to −76 dB at the center. All readings are taken using Rohde & Schwarz Vector Network Analyzer – ZNC3.

**Fig. 6.**
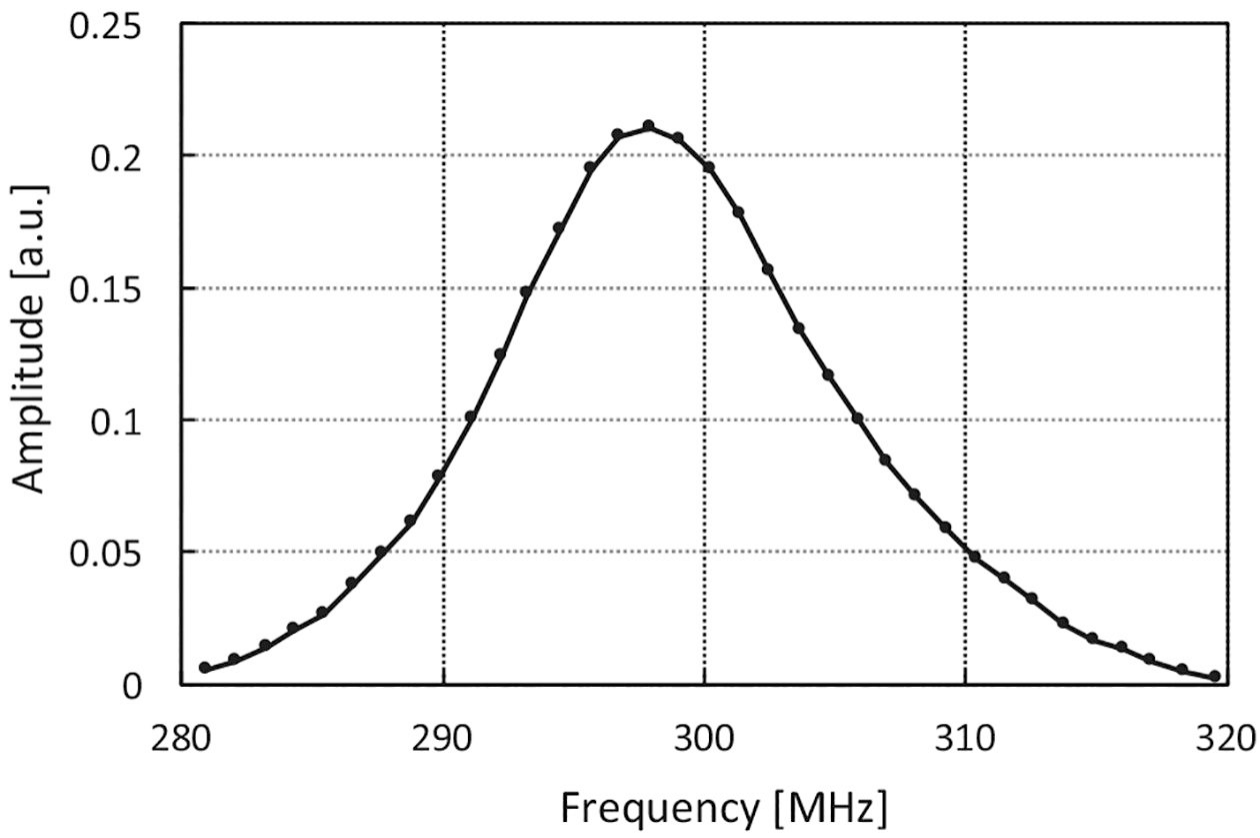
Frequency response of the coil assembly as the main field was stepped through a range to provide a resonant frequency from approximately 290–310 MHz. The RF excitation was a 30 MHz bandwidth RF pulse of 50 ns pulse width and a 10 ms pulse repetition period which was output from the Tektronix AWG 70001A.

The resonator is designed as a pair of crossed coils (Fig. 4). The outer transmit coil is a saddle coil with an inner diameter of 15 mm and a length of 25 mm. The inner receive coil is solenoidal with an inner diameter of 8 mm and positioned orthogonal to the outer coil. Both coils are tuned to 300 MHz with a Q of 25 resulting in a bandwidth of 12 MHz. This configuration provided a minimum isolation greater than 50 dB between the transmitter and receiver at resonance as shown in Fig. 5. The overall resonator bandwidth is shown in Fig. 6. A phantom consisting of a Lucite cylinder that fit snugly in the receiver coil with seven holes of different diameters was filled with solid NMP TCNQ (tetracyanoquinodimethane, an organic free radical semi-conductor with exchanged narrowed sharp single EPR resonance) for imaging.

The receive section consisted of an Agilent 8648D signal generator, a TTE KC-300 M-60 M-50–65A bandpass filter, two MiniCircuits ZX60-PL1013LN + amplifiers, and a PCIe-based Signatec PX14400A data acquisition (DAQ) board. After the EPR signal is filtered and amplified, the DAQ board digitized the signal using a 14-bit analog-to-digital converter (ADC). The signal was sampled at a clock rate of 400 MHz input from the Agilent signal generator.

A Frank sequence always contains N^2^ elements and the Hadamard sequence, contains 2^M^ elements. For direct comparison of the two sequences, a length of 256 elements was chosen for both configurations. Each RF pulse sequence consists of 256 individual pulses, centered at 300 MHz with a pulse width (PW) of 50 ns. The phase of each RF pulse is defined by the phase of each element in the corresponding pseudorandom sequence. An additional 10 ns separates the RF pulses resulting in a pulse repetition period (PRP) of 60 ns. The entire sequence cycles through every 15.36 μs. With a clock rate of 12 GB/s, the AWG requires 184,320 total points to represent the complete sequence. Low input power and high coil isolation are critical in the implementation of stochastic excitation modalities. Both contribute to the reduction in dead time which allows for a shorter PRP.

An application was developed using National Instruments Lab-VIEW to generate the data for these sequences, and transfer the data to the AWG. Once uploaded to the AWG, the desired pulse sequence is selected before conducting each experiment. For these experiments, the AWG was configured to output one of three waveforms:

1. A 300 MHz RF pulse train with a PW of 50 ns and a PRP of 20 μs.
2. A 300 MHz RF pulse train with a PW of 50 ns and a PRP of 60 ns. The pulse train is phase modulated by a 256-element biphase Hadamard sequence.
3. A 300 MHz RF pulse train with a PW of 50 ns and a PRP of 60 ns. The pulse train is phase modulated by a 256-element polyphase Frank sequence.

### 2.2. Software description

**Fig. 7.**
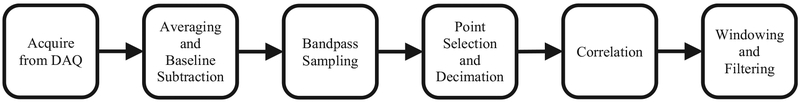
Block diagram of processing steps in the acquisition software. After acquisition, the first waveform represents the baseline. This waveform is subtracted from all other acquired waveforms. After baseline subtraction, the data is resampled using quadrature bandpass sampling. A set of 256 equally spaced points are extracted from the data and correlated with the original stochastic sequence to generate the FID. A last stage of filtering produces the final result.

A custom software application was developed to provide the following: (1) configuration of the PX14400A; (2) control and monitoring of the data acquisition process; (3) user selection of system configuration; (4) data processing (e.g., averaging, bandpass sampling, signal correlation, etc.); and (5) the graphical user interface (GUI). The GUI was developed in National Instruments LabVIEW, and uses commercially available drivers to configure and control the DAQ board. The block diagram in Fig. 7 describes the flow of the data processing portion of the program.

## 3. Results

**Fig. 8.**
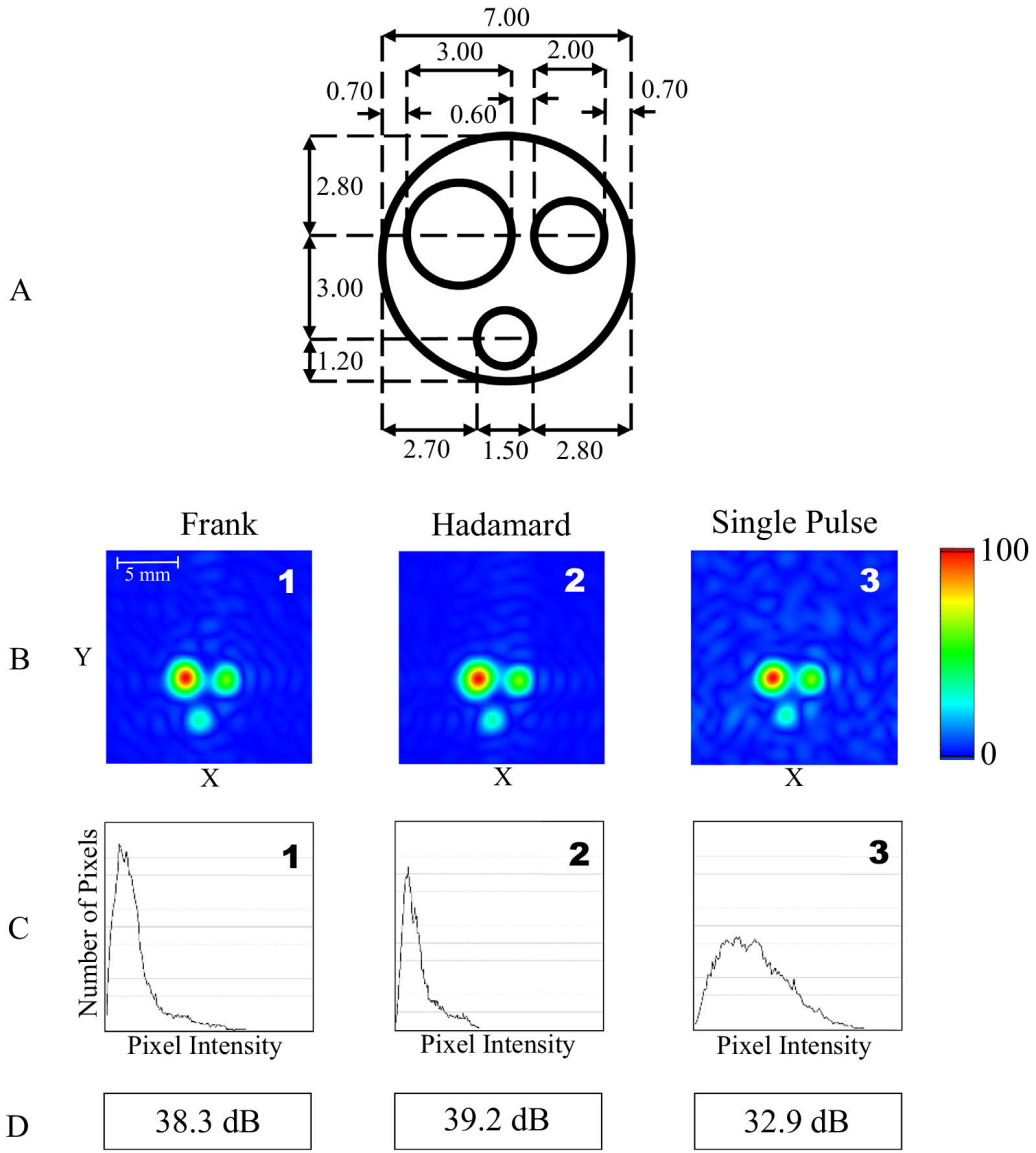
(A) Layout of the TCNQ phantom (in mm). (B) 2D images using Frank sequence encoding, Hadamard sequence encoding, and conventional single pulse system configurations using identical parameters. Gradient signals were set to 1.8 G/cm. The data was acquired as 169 projections and final images are 256 × 256 pixels. (C) Histograms of the upper half of each image to estimate background level with Frank sequence encoding, Hadamard sequence encoding, and conventional single pulse system configurations. (D) Computed signal-to-noise ratio of images (in dB) with Frank sequence encoding, Hadamard sequence encoding, and conventional single pulse system configurations.

With our system configuration (i.e., AWG used for RF pulse generation), the convolution of the RF pulse and pseudorandom sequence is completed in the custom software that creates the RF pulse sequences. Therefore, the system hardware configuration is identical for all experiments, including the single RF pulse mode. This implementation permits direct comparison of results from the different pseudorandom encoding methods and the conventional single RF pulse imaging modality. Peak RF output power was set to 5 mW in all experiments using the same resonator, corresponding to a flip angle of 0.225°. All images are obtained using phase encoding of a single time point. EPR signals were averaged in the time domain by 10,000 for each experiment. The results are summarized in Fig. 8.

## 4. Discussion

Both the Hadamard and Frank sequence imaging modalities generated images with ~40 dB SNR at RF peak power levels of 5 mW. The single pulse imaging modality at higher peak power generated images with a SNR of ~33 dB at the same peak power level. Although the Frank sequence excitation should outperform the Hadamard sequence excitation due to the systematic error in the Hadamard sequence by the imperfect circular autocorrelation, suggesting system noise rather than autocorrelation dominates the background signal. Further work to identify and minimize the impact of these sources of system noise is needed to observe the performance differences of the stochastic modalities. With the combined effects of good coil isolation and low input power, the dead time was minimized allowing the pulse repetition period to be 60 ns, enabling a 10 ns period for acquisition during the Frank and Hadamard sequence experiments. This high repetition rate resulted in a total (i.e., full duration of stochastic sequence) acquisition time of 15.36 μs. For the correlation techniques to be most efficient, the FID duration should be equal or a little less than the total acquisition time. The NMP-TCNQ probe phantom used for the experiments has T_1_ and T_2_ equal to approximately 1 μs which results in a FID duration up to approximately 5 μs, or about a third of the acquisition time. We selected a 256-element sequence for both Frank and Hadamard for comparison purposes. Based on the duration of the FID signal for this sample, a 144-element Frank sequence or a 128-element Hadamard sequence could be implemented. Image quality would remain the same, but total acquisition time would be less.

This work has demonstrated that the use of stochastic excitation and correlation detection improves SNR sufficiently in EPR imaging that average RF excitation power levels can be reduced by a factor of ~470 over traditional EPR imaging. This is an early step towards scaling up a resonator to sufficient size for a human limb or a surface coil on the head or neck region. Our future experiments will include larger phantoms and further hardware optimizations to ascertain whether we can indeed perform EPR imaging and oximetry on larger animals and humans.
